# Case Report: A novel *de novo* variant of *COL1A1* in fetal genetic osteogenesis imperfecta

**DOI:** 10.3389/fendo.2023.1267252

**Published:** 2023-11-02

**Authors:** Qiuyan Mai, Ruining Han, Yinlong Chen, Ke Shen, Shimin Wang, Qingliang Zheng

**Affiliations:** ^1^ Prenatal Diagnosis Center of the Eighth Affiliated Hospital, Sun Yat-sen University, Shenzhen, China; ^2^ Obstetrical Department of the Eighth Affiliated Hospital, Sun Yat-Sen University, Shenzhen, China

**Keywords:** osteogenesis imperfecta, *COL1A1*, *de novo*, whole exome sequencing, type I collagen

## Abstract

**Objective:**

Osteogenesis imperfecta (OI) is a rare genetic disorder. Clinical severity is heterogeneous. The purpose of this study was to investigate the genetic characteristics of a fetus with OI by whole exome sequencing (WES) and identify the cause of the disease.

**Methods:**

In this study, a fetus with osteogenic dysplasia was referred to our hospital. DNA was extracted from the aborted fetal tissue and peripheral blood of the parents. To identify the pathogenic genes, we conducted the trio-WES using DNA. A *de novo* variant in the *COL1A1* gene is suspected to be the cause of the OI phenotype. We used Sanger sequencing for validation and various bioinformatics methods (such as SIFT, PolyPhen2, Mutation Taster, conservative analysis, SWISS Model, glycosylation site prediction, and I-Mutant 2.0) for analysis.

**Results:**

Both WES and Sanger sequencing identified a novel *de novo* variant of *COL1A1* (c. 1309G>A, p. Gly437Ser) in a fetus with OI. Bioinformatic analysis showed that the affected residue, p. Gly437, was highly conserved in multiple species and predicted that the variant was deleterious and may have an impact on protein function. This variant is present in highly conserved glycine residues of Gly-X-Y sequence repeats of the triple helical region of the collagen type I α chain, which may be the cause of OI.

**Conclusion:**

This study revealed that the c.1309G>A (p. Gly437Ser) variant in the *COL1A1* gene may be the genetic cause of fetal OI in this case. The discovery of this variant enriched the variation spectrum of OI. WES improves the accurate diagnosis of fetal OI, and doctors can provide patients with appropriate genetic counseling.

## 1 Introduction

Osteogenesis imperfecta (OI) is a rare, hereditary, clinically heterogeneous, primary bone dysplasia characterized by increased bone fragility, low bone mass, and susceptibility to bone fractures. Other clinical manifestations include short stature, blue scleral hue, dentinogenesis imperfecta, progressive post-pubertal hearing loss, ligamentous laxity, and so on (1–3).

*COL1A1*-OI has been classified into four types based on clinical presentation and radiographic findings (1, 4): osteogenesis imperfecta type I (OI1) [MIM:166200], osteogenesis imperfecta type II (OI2) [MIM:166210], osteogenesis imperfecta type III (OI3) [MIM:259420], and osteogenesis imperfecta type IV (OI4) [MIM:166220]. *COL1A1*-OI is inherited in an autosomal dominant manner. The severity of *COL1A1* OI ranges from perinatal mortality to individuals with severe skeletal deformities, mobility disorders, and very short stature to nearly asymptomatic individuals with mild fracture susceptibility, normal dentition, normal stature, and normal life span (1, 3).

In Latin America, the prevalence rate of OI was 0.4/10,000 births (5). In the USA, the prevalence of OI was 0.79 per 10,000 births (6). In Argentina, the prevalence of OI was 0.34 per 10,000 births (7). A systematic review of Chinese biomedical literature related to genetic skeletal disorders published from 1978 to 2012 revealed that the second most commonly reported disease was OI (8). According to a report, the all-cause mortality hazard ratio between the OI cohort and the reference population was 2.90, indicating a higher mortality rate in patients with OI compared to the general population (9). According to relevant reports, OI is a rare genetic disease, but it can increase the mortality rate of patients with this disease. Therefore, we should pay attention to OI and achieve early detection, intervention, and appropriate treatment.

In clinical practice for prenatal diagnosis, ultrasound assessment is the first-line screening method for skeletal dysplasia in pregnancy (10). In the past, the diagnosis of OI was mostly based on clinical manifestations and related imaging examinations. In recent years, whole exome sequencing (WES) technology has been more and more applied in clinical diagnosis because of its high throughput, high accuracy, and high speed. Efficient genetic diagnostic technology is necessary for genetic counseling and the prenatal diagnosis of OI. The subdivision of OI by WES is of great significance for clinical diagnosis and treatment.

In this study, ultrasound results showed fetal osteoblastic dysplasia, suggesting that the fetus was most likely suffering from OI. To identify the genetic cause, we performed WES of fetal aborted tissue and peripheral blood from the parents, and we found that the fetus carried a novel *de novo* variant of *COL1A1* (c. 1309G>A, p. Gly437Ser). Various bioinformatics analyses showed that the variant affects protein function, indicating that the variant was most likely the cause of fetal OI.

## 2 Materials and methods

### 2.1 Ethics and informed consent

The medical study was approved by the Ethics Committee of the Eighth Affiliated Hospital of Sun Yat-sen University. The study obtained the informed consent of the proband’s parents.

### 2.2 Clinical information about the proband and parents

The subjects of the study were the proband and their parents, and the proband was a male fetus. His mother had a prenatal examination in another hospital, and the ultrasound found that the fetus was malformed (the fetal femur was short, considering osteogenic dysplasia). Therefore, the mother came to our hospital for a check-up and asked for an induced labor. Our hospital collected the medical history of the proband and family members and reexamined the ultrasound.

### 2.3 Ultrasonic examination

Ultrasound was used to confirm the prenatal diagnosis. We collected the biometric data of the fetus using a medical ultrasound device (E10, GE, USA), e.g., bi-parietal diameter (BPD), head circumference (HC), abdominal circumference (AC), femur length (FL), humerus length (HL), estimated fetal weight (EFW), etc.

### 2.4 WES

Peripheral blood of family members and aborted fetal tissue were collected, and genomic DNA was extracted using a nucleic acid extraction kit (BGI, China). The extracted DNA was determined using the ExKubit dsDNA HS assay kit (BGI, China).

The genomic DNA of the subjects was used as the detection material, and the library was constructed using the exome library construction kit (BGI, China). The detection of variants was performed using the MGISEQ-2000 (BGI, China) sequencing platform. The human genome reference sequence version selected was the UCSC hg19 Human Reference Genome. According to the standards and guidelines for the interpretation of sequence variants issued by the American College of Medical Genetics and Genomics (ACMG) in 2015 (11), the variant is classified.

### 2.5 Variant verification by Sanger sequencing

Primers were designed for candidate mutation site *COL1A1* chr17:48272452 EX20 NM_000088.3: c.1309G>A (p. Gly437Ser). The priming sequences are F: 5 '-GTAACAGCGTGAGTACCAAACTCT-3' and R: 5 '-GGGTCCTTGAACACCAACAG-3'. The DNA samples of the proband and family members were amplified by PCR. The PCR products were identified by 2% agarose gel electrophoresis and then sequenced using a sequencing instrument (3730 DNA Analyzer, ABI). The sequencing results were compared by CodonCode Aligner software (CodonCode Corporation, USA).

### 2.6 Bioinformatics analysis

The target amino acid sequences of seven species were conservatively compared using the online website COBALT (https://www.ncbi.nlm.nih.gov/tools/cobalt/). The amino acid sequence includes NP_000079.2 (*Homo sapiens*), NP_031768.2 (*Mus musculus*), NP_001003090.1 (*Canis lupus familiaris*), NP_001383551.1 (*Gallus gallus*), NP_445756.1 (*Rattus norvegicus*), XP_024835395.1 (*Bos taurus*), and NP_954684.1 (*Danio rerio*). Using the SWISS Model (https://swissmodel.expasy.org/) to predict the impact of variants on protein structure, the N- or O-glycosylation sites were predicted by NetNGlyc-1.0 (12) or NetOGlyc-4.0 (13). I-Mutant2.0 (14) was used to predict the protein stability changes of the variant, as indicated by the predicted free energy change value (DDG). A negative DDG value denotes a loss of protein stability.

## 3 Results

### 3.1 Clinical information and ultrasound findings

**Figure 1:**
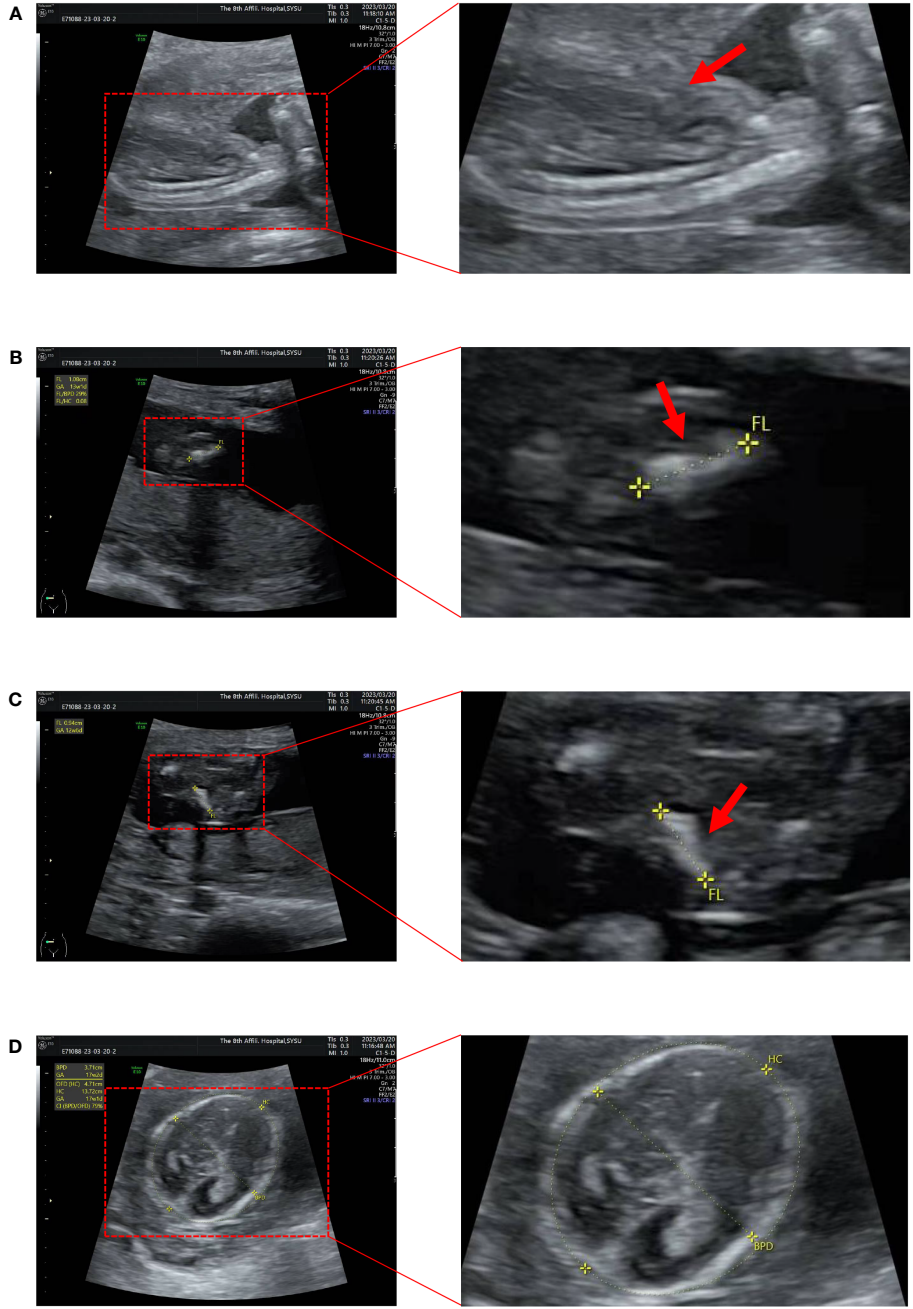
Ultrasound images of the fetus. **(A)** The thorax is narrowed and the notching can be seen at the thoracic-abdominal transition. **(B, C)** The femur is short and curved, in the shape of an earpiece. **(D)** Measure BPD and HC (BPD 37mm; HC 132mm).

A pregnant woman, aged 31 years, G1P0A0, was found to be in good health. A repeat ultrasound examination indicated that the fetus suffered from osteogenic dysplasia (**Figure 1**, **Supplementary Table 1**). Ultrasound results showed that the fetus had a BPD of 37mm, HC of 132mm, AC of 97mm, FL of 10mm, HL of 13mm, and EFW of 105 ± 15 grams. The thorax of the fetus narrowed, and the notching can be seen at the thoracic-abdominal transition of the fetus (**Figure 1A**). The limbs of the fetus showed a short and curved femur on one side, resembling an earpiece (**Figures 1B, C**). The fetus was 18 + ^5^ weeks old. However, according to the growth curves of the Asian population in the National Institute of Child Health and Human Development (NICHD) Fetal Growth Study (15), the ultrasound diagnosis was that the fetal BPD was equivalent to being 17 + ^2^ weeks old (**Figure 1D**). The fetus had short limbs, and the measured size was equivalent to being 13 + ^2^ weeks old. The physician believed that the fetus had osteogenic dysplasia.

The parents of the proband were non-consanguineous and in good health, and they denied any family history of OI. Combined with the ultrasound results of the fetus and the literature on OI, the fetus was suspected of having OI. After consultation, the woman and her family decided to terminate the pregnancy, and the appearance of the aborted fetus showed abnormal OI (no photographs were taken). Peripheral blood from family members and fetal abortion tissues were collected for trio-WES and Sanger sequencing to confirm the genetic etiology.

### 3.2 Verification results of trio-WES and Sanger sequencing

**Figure 2:**
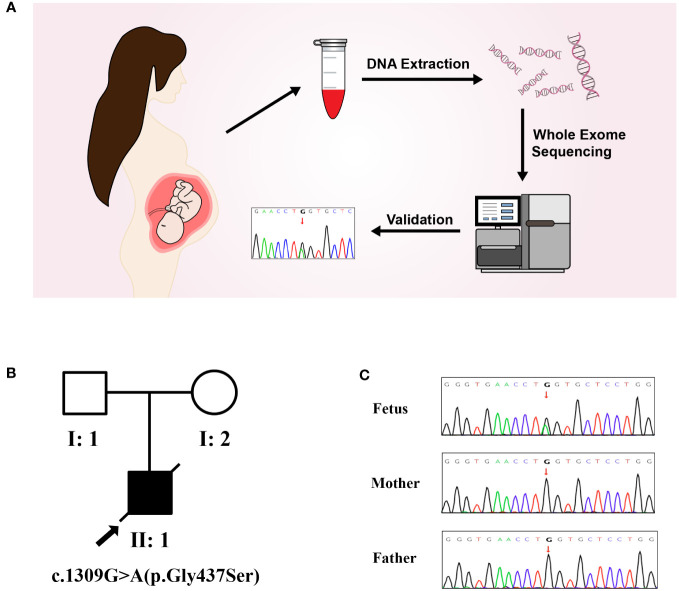
Molecular genetic analysis. **(A)** Molecular genetic analysis and validation process. **(B)** Pedigree of the family. **(C)** The mutation sites in this family were verified by Sanger sequencing. A *de novo* heterozygous variant of *COL1A1*(c.1309G>A) was found in a male fetus.

The results of trio-WES and Sanger verification found that there was a heterozygous variant c.1309G>A (p. Gly437Ser) in exon 20 of the *COL1A1* gene in the aborted fetal tissues, while this variant was not detected in the peripheral blood of the parents (**Figure 2**, **Table 1**). Combined with the results of parental detection, it is suggested that the variant is *de novo*, but the possibility that the parents are germline mosaicism carriers cannot be completely ruled out. According to the genetic variant classification standards and guidelines of the ACMG, this is categorized as a probable pathogenic variant (ACMG: PS2+PP3+PP2+PM2=LP).

**Table 1:** The mutation identified in this study.

	Nucleotide	Amino acid	Type	Status	Exon	SIFT	PolyPhen2	Mutation Taster	Father	Mother	Novel
Fetus	c.1309G>A	p. Gly437Ser	Missense	Het	20	Deleterious	Probably damaging	Disease-causing	Not detected	Not detected	Yes

After searching the Human Gene Mutation Database and the Clinvar database, it was determined that this variant had not been reported. We reported for the first time that this variant was detected in a fetus with OI.

### 3.3 Possible molecular genetic causes of fetal OI

Both WES and Sanger sequencing results showed that the fetus suspected of having OI carried a variant of c.1309G>A (p. Gly437Ser). However, this variant was not detected in the healthy parents, which is consistent with the genetic pattern. Moreover, based on the proband’s clinical information and the ACMG guidelines, we did not find any other variants in the WES test results that could explain the proband’s clinical characteristics. Therefore, the c.1309G>A of the *COL1A1* gene is very likely to cause OI in the proband.

**Figure 3:**
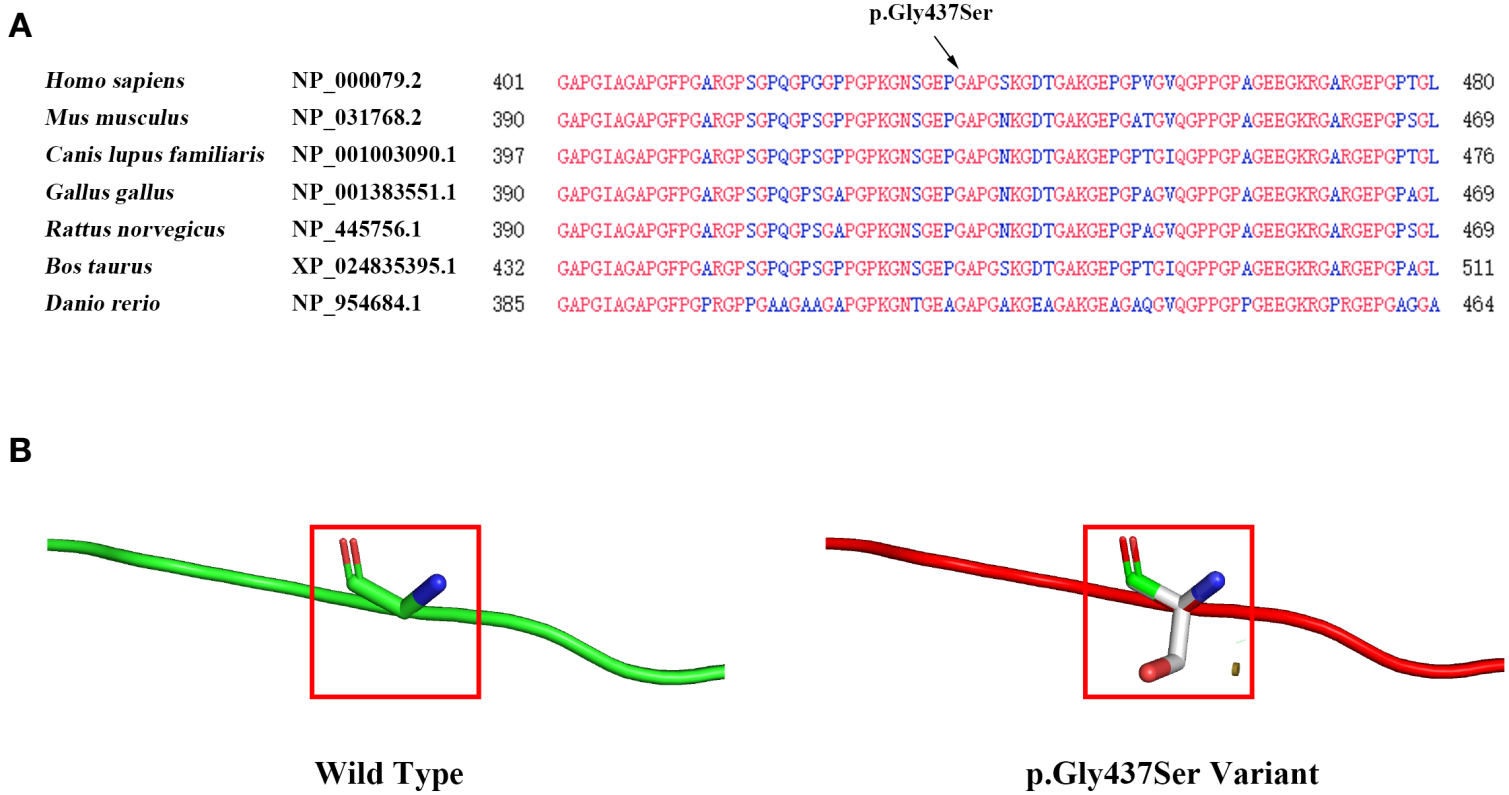
Evaluation of the influence of the*COL1A1* variant (p. Gly437Ser). **(A)** Conservation analysis showing that the p. Gly437Ser variant in *COL1A1* is conserved across *Homo sapiens*, *Mus musculus*, *Canis lupus familiaris*, *Gallus gallus*, *Rattus norvegicus*, *Bos taurus*, and *Danio rerio*. **(B)** The SWISS model predicts structural changes in the mutant protein compared to the wild-type protein.

The *COL1A1* gene encodes the collagen alpha-1 (I) chain, which contains 1464 amino acids. The p. Gly437Ser variant is located in exon 20 and the triple-helical region analyzed by the Uniprot database. The substitution of Gly by Ser will destroy the triple-helical region (Gly-X-Y) of collagen. We performed multiple sequence alignment analyses using COBALT. We found that Gly is highly conserved in seven species (*Homo sapiens*, *Mus musculus*, *Canis lupus familiaris*, *Gallus gallus*, *Rattus norvegicus*, *Bos taurus*, and *Danio rerio*), suggesting that amino acid substitution at this site is likely to affect the protein (**Figure 3A**). Moreover, the bioinformatics software SIFT, PolyPhen2, and Mutation Taster predicted this p. Gly437Ser variant to be deleterious (**Table 1**). In addition, we used the SWISS Model, a simulation program for bioanalysis, to predict the three-dimensional structure of the mutant protein. We observed that p. Gly437Ser resulted in an extra side chain, which may affect the binding ability of the collagen chain and disrupt the function of the protein (**Figure 3B**).

NetNGlyc-1.0 and NetOGlyc-4.0 servers predicted that *COL1A1* may have one possible N-glycosylation site (position: 1365) and six possible O-glycosylation sites (position: 418, 441, 546, 553, 936, 1146), while the variant (p. Gly437Ser) of *COL1A1* may result in one possible N-glycosylation site (position: 1365) and five possible O-glycosylation sites (position: 184, 492, 541, 1069, 1193) (**Supplementary Table 2**). Meanwhile, we found that the p. Gly437Ser variant was predicted to decrease protein stability (**Supplementary Table 2**). All these analyses indicated that the p.Gly437Ser variant may affect the 3D structure and post-translational modification of *COL1A1*. We speculate that these changes in *COL1A1* may affect the interaction between *COL1A1* and the cell receptors (which include members of the integrin family, Endo180, the tyrosine kinase Discoidin Domain Receptors, and the Leukocyte-Associated Immunoglobulin-like Receptor (16), finally leading to the occurrence of OI.

## 4 Discussion

*COL1A1* is located on chromosome 17q21.33 and consists of 51 exons (17, 18). *COL1A1* is a gene that is responsible for encoding the alpha-1 chain of type I collagen, which is the most abundant protein in mammals (19). Type I collagen is mainly secreted by osteoblasts, dermal fibroblasts, and tenocytes (20). The basic functional unit of type I collagen is a heterotrimer consisting of two α1 chains and one α2 chain (which is produced from the *COL1A2* gene) that assemble together to form a triple helical structure (19, 21). The occurrence of OI caused by the *COL1A1* gene generally includes two classes of mutations: one causes quantitative defects, and the other causes the synthesis of collagen molecules with structural abnormalities (qualitative defects) (22). According to literature reports, mutations that cause quantitative defects may lead to milder phenotypes in patients and are associated with OI type I (20, 23). In contrast, mutations that cause qualitative defects can lead to more severe phenotypes in patients and are associated with moderate and progressive deforming OI type IV and OI type III, in addition to lethal OI type II (20, 23). The most common mutation that leads to structural collagen defects is single nucleotide mutations, in which a bulkier or charged residue replaces glycine in the helical domain (Gly-X-Y) of type 1 collagen (20, 23).

A total of 500 variants were detected in WES in the proband and included 38 variants of skeletal system diseases. However, most of the variants were not related to the patient’s clinical manifestations. We conducted a specific analysis based on the clinical manifestations of the proband, including OI and bone dysplasia. Among them, there were four variants related to OI and six variants related to bone dysplasia. Among them, six were rated as likely benign and could be excluded. The other four heterozygous variants included two that had uncertain significance (*CILK1* chr6:52883264 EX8 NM_016513.4: c.527A>G (p. Asn176Ser); *CRTAP* chr3:33156016 EX1 NM_006371.4: c.447G>A (p. Lys149=)) and two likely pathogenic variants (*TYROBP* chr19:36398457-36398458 EX3 NM_003332.3: c.119dup (p. Val42Argfs*45); *COL1A1* chr17:48272452 EX20 NM_000088.3: c.1309G>A (p. Gly437Ser)). However, the three variants corresponding to *CILK1*, *CRTAP*, and *TYROBP* were all detected in the proband’s father, who had no clinical manifestations of OI. Furthermore, the clinical manifestations of the diseases caused by *CILK1* and *TYROBP* were not consistent with the clinical features of the patient. Moreover, the variant of *CRTAP* is a synonymous mutation, which is predicted by software to have no effect on splicing and has not been reported in the literature. Therefore, these three variants were not the cause of the proband’s disease. Another variant of *COL1A1* was only detected in the proband and not in the normal parents. Moreover, the pathogenic variant of *COL1A1* can cause OI in autosomal dominant inheritance. This suggests that the variant is consistent with the patient’s clinical manifestations and genetic pattern. Interestingly, a study reported a male infant with an intrauterine fracture resembling OI who tested negative for *COL1A1* and *COL1A2* mutations. In that case, the patient was discovered to have a pathogenic homozygous mutation in the *CCDC134* gene, which is associated with fragility fractures (24). In order to avoid missing the detection of variants, we also focused on genes related to OI other than *COL1A1* and *COL1A2*. However, we did not find *CCDC134*-related variants in this proband, and the variants in other OI genes did not fit with the clinical manifestations and inheritance pattern of this patient. In conclusion, the variant of *COL1A1* (p. Gly437Ser) is the only variant that could explain the OI of the proband.

In this study, we identified a *de novo* heterozygous variant (c. 1309G>A, p. Gly437Ser) of the *COL1A1* gene in a fetus with OI for the first time. The ultrasound results at 18 + ^5^ gestational weeks showed that the fetus had osteogenic dysplasia and a narrowed thorax, which indicated that the fetus was most likely suffering from type II OI. Type II OI has a more severe phenotype and can be detected by ultrasound in the second trimester of pregnancy (25). OI type II is a fatal type of OI characterized by bone fragility, with many perinatal fractures, severe bowing of the long bones, undermineralization, and death in the perinatal period due to respiratory failure (1, 26). A study of 146 OI patients from different populations showed that 56.16% of cases of OI were caused by *de novo* pathogenic variants, the majority of *de novo* cases were missense pathogenic variants, and 61.54% of Gly to Ser substitutions occurred in *de novo* cases (27). Moreover, Lidiia Zhytnik and collaborators found that *de novo* OI cases were characterized by a high prevalence of collagen qualitative defects, whereas hereditary OI cases were characterized by quantitative collagen defects (27). The *de novo* variant in our study is also a Gly to Ser substitution, which is a qualitative defect that causes changes in the structure of collagen. 

p. Gly437Ser is located in the triple helix region of the *COL1A1* gene. Amino acid conservation analysis showed that the amino acid at position 437 was highly conserved in different species. The variant changes the glycine at 437 to serine, disrupting the formation of the triple helix structure. Moreover, bioinformatics analysis predicted that this variant would lead to the addition of side chains, which could affect the binding ability of collagen chains. The glycosylation modification was also changed, and the protein stability was predicted to decrease, which would affect the function of the protein. These bioinformatics analysis results indicate that the variant may lead to structural abnormalities in collagen molecular synthesis, which may lead to more severe phenotypes. Studies have shown that glycine to valine substitutions in the Gly-X-Y triplets, from glycine 256 to glycine 1006, of the triple helical domain of type 1 collagen chains, produce the OI type II phenotype (28, 29). In addition, it has been reported that p. Gly425Ser, which is adjacent to the variant in this case (p. Gly437Ser), has been detected in patients with OI type II, and p. Gly425Ser is also located in the triple helix region (30). These previous studies also reiterated the importance of the triple helix region of the *COL1A1* gene in maintaining its protein function and that the missense mutation of glycine in the triple helix region is closely related to OI type II.

In conclusion, this study confirmed the heterozygous variant of the *COL1A1* gene (c. 1309G>A, p. Gly437Ser) as the pathogenic cause of fetal OI in this case by WES and Sanger sequencing, which enriched the OI mutation spectrum and phenotype spectrum caused by the *COL1A1* gene mutation. This provides the basis for family genetic counseling and reproductive guidance. The improvement of the phenotypic evaluation of the fetus and family members, combined with gene variation analysis, is of great significance for determining the pathogenicity of rare mutations and fetal prognosis. Understanding genetic defects also increases the prospects for targeted therapies. Moreover, the use of WES can help physicians genetically classify patients. Unlike clinical classification, the genetic type of an individual does not change with age or between family members.

## Data availability statement

The original contributions presented in the study are included in the article/**Supplementary Material**. Further inquiries can be directed to the corresponding author.

## Ethics statement

The studies involving humans were approved by Ethics Committee of the Eighth Affiliated Hospital of Sun Yat-sen University. The studies were conducted in accordance with the local legislation and institutional requirements. Written informed consent for participation in this study was provided by the participants’ legal guardians/next of kin. Written informed consent was obtained from the individual(s), and minor(s)’ legal guardian/next of kin, for the publication of any potentially identifiable images or data included in this article.

## Author contributions

QM: Conceptualization, Data curation, Formal analysis, Writing – original draft, Writing – review & editing. RH: Conceptualization, Data curation, Investigation, Writing – review & editing. YC: Data curation, Formal analysis, Writing – original draft. KS: Investigation, Writing – original draft. SW: Data curation, Investigation, Writing – original draft. QZ: Conceptualization, Funding acquisition, Project administration, Writing – review & editing.
