# Supplementary material for: Case Report: A novel de novo variant of COL1A1 in fetal genetic osteogenesis imperfecta
Source: Front Endocrinol (Lausanne). 2023 Nov 2;14:1267252. doi: 10.3389/fendo.2023.1267252 (PMC10653333; doi:10.3389/fendo.2023.1267252)
Supplement: Supplementary file 1 [file Table_1.docx]

**Supplementary Table 1 Ultrasound measurements of the fetus at 18^+5^ weeks**

|  | BDP (mm) | AC (mm) | FL (mm) | HC (mm) | HL (mm) | EFW (g) |
| --- | --- | --- | --- | --- | --- | --- |
| Reference* | 18 weeks: 35.8 - 44.7  19 weeks: 38.7 - 47.9 | 18 weeks: 114.5 - 139.4  19 weeks: 125.5 - 151.8 | 18 weeks: 21.9 - 29.5  19 weeks: 24.6 - 32.4 | 18 weeks: 135.6 - 160.4  19 weeks: 146.8 - 172.3 | 18 weeks: 22.0 - 29.0  19 weeks: 24.3 - 31.5 | 18 weeks: 177 - 261  19 weeks: 217 - 321 |
| Fetus | 37 | 97 | 10 | 132 | 13 | 105±15 |

* Asian population of NICHD Fetal Growth Study(Buck Louis et al., 2015) as a reference (5^th^-95^th^ percentile).

**Supplementary Table 2 The N- or O-glycosylation sites and DDG value of p. Gly437Ser**

|  | N-glycosylation sites (position) | O-glycosylation sites (position) | DDG (Kcal/mol) |
| --- | --- | --- | --- |
| Wild Type | 1365 | 418, 441, 546, 553, 936, 1146 | - |
| p. Gly437Ser | 1365 | 184, 492, 541, 1069, 1193 | -0.06 |
